# General practice referral of ‘at risk’ populations to community leisure services: applying the RE-AIM framework to evaluate the impact of a community-based physical activity programme for inactive adults with long-term conditions

**DOI:** 10.1186/s12889-019-7701-5

**Published:** 2019-10-17

**Authors:** E. L. Bird, M. S. Y. Biddle, J. E. Powell

**Affiliations:** Centre for Public Health and Wellbeing, University of the West of England, Frenchay Campus, Bristol, BS16 1QY UK

**Keywords:** Physical activity, Referral, Social prescribing, Long-term conditions, Implementation, Evaluation, RE-AIM

## Abstract

**Background:**

In the UK a high proportion of adults with long-term conditions do not engage in regular physical activity. General practice (GP) referral to community-based physical activity is one strategy that has gained traction in recent years. However, evidence for the real-world effectiveness and translation of such programmes is limited. This study aimed to evaluate the individual and organisational impacts of the ‘CLICK into Activity’ programme - GP referral of inactive adults living with (or at risk of) long-term conditions to community-based physical activity.

**Methods:**

A mixed methods evaluation using the RE-AIM framework was conducted with data obtained from a range of sources: follow-up questionnaires, qualitative interviews, and programme-related documentation, including programme cost data. Triangulation methods were used to analyse data, with findings synthesised across each dimension of the RE-AIM framework.

**Results:**

A total of 602 individuals were referred to CLICK into Activity physical activity sessions. Of those referred, 326 individuals participated in at least one session; the programme therefore reached 30.2% of the 1080 recruitment target. A range of individual-, social-, and environmental-level factors contributed to initial physical activity participation. Positive changes over time in physical activity and other outcomes assessed were observed among participants. Programme adoption at GP surgeries was successful, but the GP referral process was not consistently implemented across sites. Physical activity sessions were successfully implemented, with programme deliverers and group-based delivery identified as having an influential effect on programme outcomes. Changes to physical activity session content were made in response to participant feedback. CLICK into Activity cost £175,000 over 3 years, with an average cost per person attending at least one programme session of £535.

**Conclusions:**

Despite not reaching its recruitment target, CLICK into Activity was successfully adopted. Positive outcomes were associated with participation, although low 6- and 12-month follow-up response rates limit understanding of longer-term programme effects. Contextual and individual factors, which may facilitate successful implementation with the target population, were identified. Findings highlight strategies to be explored in future development and implementation of GP referral to community-based physical activity programmes targeting inactive adults living with (or at risk of) long-term conditions.

## Background

Physical activity is associated with a range of positive physical and mental health outcomes, including a reduced risk of heart disease, obesity, type 2 diabetes, and improved emotional wellbeing [1, 2]. However, despite clear evidence demonstrating the benefits associated with regular physical activity, self-report data from England suggest that approximately one fifth of men and one quarter of women are inactive (defined as participation in less than 30 min per week of moderate physical activity, or less than 15 min per week of vigorous physical activity), [3] costing an estimated £7.4 billion each year [4]. Compared with the general UK population, physical inactivity prevalence is even higher among those living with a long-term condition (such as type 2 diabetes and hypertension) [5]. Effective physical activity promotion strategies are therefore required to reduce unsustainable pressure on local health and social care provision [6, 7].

An increase in long-term conditions, alongside an ageing population, has created pressure on the delivery of services in General Practice (GP). This has led to General Practitioners and commissioners in the UK advocating and developing collaborative working practices with social prescribing services in the community [8]. Social prescribing schemes allow primary health care professionals to refer patients to a non-medical service, such as community-based physical activities, art classes, and nature-based activities, with the aim of improving patients’ health and wellbeing [8, 9].

The benefits of group-based physical activity for those living with long-term conditions are well established, [10, 11] and this has resulted in group-based physical activity becoming one of the main activities of social prescription made through GP referral to leisure facilities in the community [8]. However, despite increased interest in social prescribing via GP referral to physical activities delivered in leisure centres, two recent systematic reviews of UK-based social prescribing revealed limited evidence for its effectiveness, with differences in methodological quality and reporting standards identified [9, 12]. Furthermore, there is little evidence for the real-world effectiveness and translation of group-based physical activity programmes specifically targeted at inactive adults with long-term conditions. The most recent systematic review evidence on this topic combine evidence from active and inactive adults, [13] and healthy and unhealthy populations [14]. As such, effort is required to better understand the individual and contextual factors that may facilitate the successful real-world delivery of such programmes, and to generate evidence on their cost and long-term sustainability [15, 16].

Review-level evidence suggests that the implementation of effective and sustainable physical activity programmes requires improved transparency and consistency of reporting, [13, 16] and further consideration of programme external validity [16]. One framework for evaluating public health programmes that has gained attention in recent years is the Reach, Effectiveness, Adoption, Implementation and Maintenance (RE-AIM) framework [17]. RE-AIM is a multi-level framework that aims to measure the effects of complex interventions while also identifying the barriers and facilitators to real-world intervention implementation [18]. It has five dimensions which identify factors influencing internal and external validity: *Reach* of the intervention for the target population; *Effectiveness* of the intervention on desired outcomes; *Adoption* of the intervention at setting and staff levels; *Implementation*, delivery of intervention as intended and participant adherence; *Maintenance* of intervention effects over time, at individual and organisational levels [17].

CLICK into Activity was a physical activity programme that aimed to support inactive adults living with at least one of the following conditions: type 2 diabetes, pre-diabetes (defined as those with higher than normal glucose levels, but not meeting criteria for type 2 diabetes), hypertension, and overweight or obesity, to increase physical activity levels. It is an example of social prescribing, with individuals referred to community-based physical activity by a primary health care professional. The overall aim of this study was to evaluate the individual and organisational impacts of CLICK into Activity.

## Methods

### CLICK into activity

A district council located in a rural part of South West England led the CLICK into Activity programme. It was funded by Sport England for 3 years from September 2015 with matched funding support from local partners. A project management team (including members of the district council, local partners and Sport England) developed the programme, including individual and organisational recruitment targets and programme content. The authors were not involved in the programme development phase; involvement was limited to evaluation of the programme.

Gatekeepers from nine GP surgeries agreed to participate in GP referral to community-based physical activity, and three trained exercise specialists experienced in working with inactive adults were recruited to deliver the physical activity sessions. At the time of programme delivery there was no local provision for GP referral that met the UK’s recommendations for promoting physical activity among those with long-term conditions [19].

Initially, patients diagnosed with type 2 diabetes, pre-diabetes and/or hypertension and registered at a participating GP surgery were referred to community-based physical activity through one of two channels: 1) Direct contact with a General Practitioner or health care professional during a routine appointment or, 2) GP surgery mail-out to potentially eligible patients based on medical records. Eligibility to participate in the programme was also determined by physical activity levels; only those classified as ‘inactive’ (individuals reporting total physical activity of 30 or fewer minutes per week) [20] were invited to attend. Early participant recruitment to the programme was low, and so the eligibility criteria were relaxed in December 2017 to include obese and overweight individuals (with a body mass index (BMI) > 25 kg/m^2^).

During an initial consultation with an exercise specialist, eligible participants had the opportunity to discuss suitable physical activity options and were encouraged to attend at least one physical activity session per week, but there was no minimum or maximum number of sessions that each participant could attend during their 12-week enrolment period. The physical activity sessions were delivered by exercise specialists, trained in motivational interviewing, in local leisure centres and other community venues, and offered a range of group-based activities (i.e. sessions attended by two or more individuals) in response to evidence supporting group-based physical activity promotion [11, 12]. Sessions were delivered at various times throughout the week, and included circuit training, walking groups, and adapted sports (e.g. seated volleyball). The project management group used local long-term conditions data held by participating surgeries to set a recruitment target for participants. The project aimed to support 1080 participants to attend and participate in at least one 30-min CLICK into Activity session during the 12-week enrolment period.

A logic model, presenting the programme delivery plan and hypothesised outcomes can be found in Additional file 1.

### Study design

To generate evidence on each dimension of the RE-AIM framework a mixed methods approach was utilised. CLICK into Activity participants provided individual-level data, while organisational-level data were collected from members of the project steering group and the exercise specialists responsible for delivering the programme. Consistent with methods utilised in previous applications of the RE-AIM framework in the area of physical activity promotion, [21, 22] data were obtained from a range of sources: questionnaires, qualitative interviews, and programme-related documentation (Table 1). Evaluation data were collected between September 2015 and June 2018.

**Table 1:** Overview of data sources and their contribution to RE-AIM dimensions

Data source	RE-AIM dimension				
	Reach	Effectiveness	Adoption	Implementation	Maintenance
Questionnaires	✓	✓			✓
Semi-structured interviews	✓	✓	✓	✓	✓
Programme-related documentation	✓		✓	✓	

*Reach* of the intervention for the target population; *Effectiveness* of the intervention on desired outcomes; *Adoption* of the intervention at setting and staff levels; *Implementation*, delivery of intervention as intended and participant adherence; *Maintenance* of intervention effects over time, at individual and organisational levels [17]

### Participant recruitment

This study was granted ethical approval by the University of the West of England Ethics Committee. All participants were provided with information about the evaluation and gave written informed consent to participate.

#### CLICK into activity participants

All adults referred to CLICK into Activity were invited to participate in the evaluation at an initial GP surgery consultation with a trained exercise specialist. Data were collected via self-report questionnaires and objective measures administered at baseline (T0), immediately after the 12-week programme (i.e. 3-month follow-up) (T1), 6-month follow-up (T2) and, 12 months after programme completion (T3). Data collection measures were the same at each time point. An exercise specialist conducted T0 data collection during the initial consultation. Follow-up data collection was conducted by an exercise specialist at participating GP surgeries, where possible, or via telephone appointment at the request of a participant. The follow-up data collection mode was not recorded. Each participant was assigned a unique serial number to ensure participant anonymity and to match responses at each data collection point.

#### Interview participants

A range of programme stakeholders, including the CLICK into Activity programme manager, a practising General Practitioner (and member of the project steering group), the exercise specialists responsible for delivery, and CLICK into Activity participants were invited to participate in a 30-min semi-structured telephone interview. Organisational-level stakeholders were invited opportunistically during a project steering group meeting. Telephone interviews with exercise specialists took place approximately 1 year and 2 years into programme delivery, with the programme manager and General Practitioner participating in an interview approximately 2 years into programme delivery. CLICK into Activity participants that agreed to be contacted for interview during initial consultation were invited to participate, with the first ten available recruited to the qualitative aspect of the evaluation. The objective of conducting interviews with multiple stakeholders throughout programme delivery was to gather evidence that could contribute to all dimensions of the RE-AIM framework.

### Measures

#### Questionnaires

Self-reported questionnaires assessed weekly physical activity via the short-form International Physical Activity Questionnaire (IPAQ short), [23] which included time spent engaged in moderate and vigorous physical activity, time spent walking and sitting time. Self-reported participation in sport was assessed using the Single Item Sport England Measure: “During the last 7 days, on how many days did you take part in any sport?” (days per week) followed by “How much time did you usually spend doing sport on one of those days?” (hours/minutes per day). Mental wellbeing was assessed using the validated 14-item Warwick Edinburgh Mental Wellbeing Scale (WEMWBS) [24]. Participants were asked to respond to each item, for example “I’ve been feeling optimistic about the future”, on a 5-point Likert scale from 1 (None of the time) to 5 (All of the time). Participants’ socio-demographic characteristics (sex, age, ethnicity, health status, education, annual income and relationship status) were also captured. To calculate BMI, an exercise specialist at each time point objectively measured height and weight. Low muscle strength has been linked to higher risk of premature death, [25] and as such, exercise specialists used a dynamometer to measure participant grip strength at each time point. Participants completing follow-up measures via telephone were unable to provide objective grip strength, and height and weight data. For an overview of participant characteristics, please see Table 2.

**Table 2:** Comparison of participant characteristics at baseline according to programme participation

Characteristic	Total sample (*N* = 602)		Participants (*N* = 326) ^a^		Non-participants (*N* = 276) ^a^		*p* ^*b*^
	*N*	*%*	*N*	*%*	*N*	*%*	
Sex							
Female	379	63.0	204	62.6	174	63.3	0.87
Age							
18–34	17	2.8	6	1.8	11	4.0	0.01
35–50	42	7.0	14	4.3	28	10.2	
51–69	229	38.0	125	38.3	104	37.8	
70 or above	309	51.3	180	55.2	128	46.5	
Body Mass Index (BMI)							
25 or under	52	8.6	34	10.4	18	6.5	0.20
26–29	134	22.3	70	21.5	64	23.3	
30 or above	369	61.3	193	59.2	175	63.6	
Ethnicity							
White	580	96.3	318	97.5	261	94.9	0.84
Mixed ethnic group	3	0.5	1	0.3	0	0.0	
Black British	1	0.2	1	0.3	2	0.7	
Asian	2	0.3	1	0.3	1	0.4	
Asian British	2	0.3	1	0.3	1	0.4	
Other	4	0.7	1	0.3	3	1.1	
Education							
Degree/degree level qualification	128	21.3	76	23.3	52	18.9	0.15
A level or equivalent	50	8.3	24	7.4	26	9.5	
Professional qualification^c^	166	27.6	77	23.6	88	32.0	
O level passes/GCSE passes or equivalent	91	15.1	48	14.7	43	15.6	
CSE/SCE	32	5.3	15	4.6	17	6.2	
Other	18	3.0	15	4.6	3	1.1	
No qualifications	108	17.9	68	20.9	40	14.5	
Annual household income							
Up to £9999	84	14.0	38	11.7	46	16.7	0.63
£10,000 - £19,999	177	29.4	112	34.4	65	23.6	
£20,000 - £29,999	77	12.8	46	14.1	31	11.3	
£30,000 - £39,999	41	6.8	15	4.6	26	9.5	
£40,000 - £49,999	23	3.8	12	3.7	11	4.0	
£50,000 or above	28	4.7	11	3.4	17	6.2	
Don’t know	99	16.4	59	18.1	40	14.5	
Prefer not to say	65	10.8	32	9.8	32	11.6	
Relationship status							
Single	54	9.0	26	8.0	28	10.2	0.33
Have partner but do not live together	7	1.2	3	0.9	4	1.5	
Live with partner	47	7.8	25	7.7	22	8.0	
Married and live with partner	340	56.5	192	58.9	147	53.5	
Married and separated from partner	21	3.5	9	2.8	12	4.4	
Divorced	47	7.8	21	6.4	26	9.5	
Widowed	79	13.1	49	15.0	30	10.9	

^a^Participants defined as those who attended at least one CLICK into Activity session during the 12-week programme

^b^Comparison of those who attended at least one CLICK into Activity session during the 12-week programme (‘participants’) with those who did not (‘non-participants’)

^c^Equivalent to Nursing, Midwifery, City and Guilds qualifications

#### Semi-structured interviews

Qualitative methods were employed by ELB and MB to elicit in-depth feedback on CLICK into Activity, to better understand its impact on physical activity outcomes and to reflect upon implementation (from GP referral to CLICK into Activity sessions), including positive and negative aspects of the programme. A semi-structured interview schedule was developed to explore each of the dimensions of the RE-AIM framework from the perspective of each stakeholder group (CLICK into Activity participants; exercise specialists; organisational-level stakeholders). For example, initial interviews with CLICK into Activity participants and exercise specialists explored individual and organisational programme effectiveness; secondary interviews, conducted with the same individuals approximately one-year later, provided an opportunity to reflect upon earlier responses and discuss individual and organisational programme maintenance. An example interview schedule used in the first interview with CLICK into Activity participants is presented in Additional file 2.

#### Programme-related documentation

Exercise specialists recorded participant attendance for the duration of the programme using a paper-based register. These data were entered into an Excel spreadsheet and were used to assess programme participation (participants that attended at least one CLICK into Activity session) and average attendance. Data on resource use and actual costs incurred between programme initiation (September 2015) and June 2018 were provided by the programme manager and used to populate a resource use checklist adapted from previous studies [26, 27].

### Data analyses

Questionnaire data were entered into IBM SPSS Statistics (v.22.0). IPAQ questionnaire data were cleaned in line with IPAQ guidelines [28]. Descriptive statistics were generated for each outcome of interest at each time point. Linear mixed models for repeated measures over time were used to assess whether CLICK into Activity had an effect on participants’ physical activity, mental wellbeing and grip strength outcomes, with fixed effects of time and initial participation in at least one CLICK into Activity session. Models were adjusted for baseline socio-demographic characteristics (age, sex, BMI) and GP surgery. This approach was used to account for repeated measures over time and to prevent listwise deletion due to missing data [29]. Data were found not to be missing completely at random (Little’s MCAR test, *p* = 0.00) and subsequent missing value analysis indicated that data were missing at random. Bonferroni adjusted post hoc tests compared outcomes to each time point (baseline, 3-month follow-up, 6-month follow-up and 12-month follow-up) to estimate means and 95% confidence intervals (95% CI) for changes in outcomes.

Qualitative audio data were transcribed verbatim and analysed using NVivo 10 (QSR International). Data were explored using thematic analysis, [30] with the coding process based predominantly on mapping data against each of the RE-AIM dimensions in line with recently published guidance [31]. Analysis aimed to generate a balanced assessment of the programme and the factors that may have had an impact on the reach, effectiveness, adoption, implementation and potential sustainability of CLICK into Activity. Data saturation was reached when further coding was no longer feasible [32]. To confirm accuracy and interpretation of the data during the coding process and at theme development, findings were discussed and agreed between authors and reported in line with COREQ guidelines (see Additional file 3, [33]).

Programme cost data were categorised according to stage of programme delivery as follows: programme preparation cost estimate; programme delivery cost estimate; and, programme research and development cost estimate, to reflect the actual mainstream costs of the programme in a real-world delivery scenario.

### Data synthesis

Triangulation methods [34] were utilised to explore complementary and concurrently collected quantitative and qualitative data, considering evidence from different perspectives with the aim of developing a comprehensive understanding of the population impact of CLICK into Activity, from GP referral to CLICK into Activity session delivery. Consistent with previously utilised methods, [22] data from all sources were synthesised across each dimension of the RE-AIM framework. Programme reach, effectiveness and maintenance were assessed quantitatively using self-report and objectively measured data provided by CLICK into Activity participants. Evidence pertaining to effectiveness and maintenance domains is presented together in response to the quantitative analysis approach undertaken. Semi-structured interview data from a range of programme stakeholders were used to provide insight into factors influencing each dimension of the RE-AIM framework. Programme-related documentation, including attendance records and cost data, provided evidence for programme reach, adoption, and implementation.

## Results

A total of 621 individuals completed baseline measures, with 602 found to be eligible to participate in the CLICK into Activity programme. Three-month follow-up measures were completed by 186 participants (30.9%), followed by 80 participants at 6-months (13.3%), with 41 participants completing a 12-month follow-up questionnaire (6.8%).

Twenty-seven telephone interviews were conducted. One interview was conducted with the CLICK into Activity programme manager, and one with a General Practitioner (and member of the project steering group). All three CLICK into Activity exercise specialists took part in two interviews (1 year and 2 years into project delivery). Ten CLICK into Activity participants took part in an interview soon after completion of the 12-week programme, with nine completing a second interview 1-year post-CLICK into Activity enrolment. One participant emigrated and was unavailable for a second interview. Eight participants attended at least one physical activity session each week during their 12-week enrolment, one had attended 9 sessions, and one participated in 6 sessions. Results corresponding to each of the RE-AIM dimensions are presented below.

### Reach

Of those eligible to participate in CLICK into Activity (*N* = 602), the majority were referred to the programme due to diagnosis of pre-diabetes, type 2 diabetes or hypertension (*N* = 558, 92.6%). Following relaxation of the eligibility criteria in December 2017, 22 participants were referred due to being obese or overweight (3.7%), and 22 were referred for having a combination of long-term conditions (3.7%). Table 2 reveals that the majority of participants were female (*N* = 379, 63.0%) and just over half of participants were aged 70 or above (*N* = 309, 51.3%). Most participants identified as being of White ethnic origin (*N* = 580, 96.3%) and just over one fifth were qualified to degree level (or equivalent) (*N* = 128, 21.3%). Almost one third of participants reported an annual household income of between £10,000 and £20,000 (*N* = 177, 29.4%), and most participants described themselves as being in a relationship (*N* = 394, 65.5%). More than 80% of respondents had a baseline BMI within the overweight and obese categories (BMI ≥25 kg/m^2^, *N* = 503, 83.6%).

A range of communication strategies were employed to reach the target audience, including newspaper advertising, display screens at participating GP surgeries, local community sites (online and print), and flyer distribution by exercise specialists in the local area, all advertising the programme. Despite utilising various communication channels, recruitment was low at the outset of the project. However, approximately 18 months into project delivery GP surgeries agreed to a mail-out to all patients diagnosed with at least one of the long-term conditions targeted by the programme. Recruitment figures were seen to increase following mail-outs, and qualitative interviews suggested that a wide ranging and long-term promotional strategy was essential.

I think continual promotion [is important] because there can be a lot of promotion at the beginning of the project, but then not so much as you get into the project. There hasn’t been continual promotion with the practice managers. You can’t just do [promotional activities] once and expect it to filter through to everybody. *Exercise specialist 3*

CLICK into Activity aimed to support 1080 individuals to attend and participate in at least one 30-min physical activity session during the 12-week programme. Of the 602 eligible individuals that provided baseline data individuals that provided baseline data, attendance records revealed that 326 participants attended at least one 30-min session (30.2% of 1080 target population). These participants attended nine sessions, on average, during the 12-week programme (M = 8.6, SD = 6.0). Just over one third of participants attended at least 12 sessions during the 12-week enrolment period (*N* = 104, 32%). These figures suggest that CLICK into Activity sessions were well-liked by participants in that once they had attended one session they often returned for more. As shown in Table 2 analysis identified no differences in sex, ethnicity, relationship status or baseline BMI between those that attended a CLICK into Activity session and those that did not (*p* > 0.05). There was a significantly higher proportion of those aged 70 or above attending a session compared with those that did not (55.2% vs 46.5%, respectively; *p* = 0.01).

Interviews with stakeholder groups identified a range of barriers and facilitators influencing initial participation in a physical activity session. For example, individual-level factors such as personal motivation to lose weight, concerns regarding potential for exacerbating existing health issues, and the fear of embarrassment, were noted. Wider social- and environmental-level factors, including activity scheduling and social support from family, friends and the community were also identified. Qualitative interviews also highlighted the importance of the physical environment, for example the influence of the rural setting on perceptions of neighbourhood safety and subsequent participation.

In rural Somerset the evening sessions were not successful, and that was down to the target population not feeling confident going out when it was dark. So, when the winter came, and the nights were drawing in people weren’t feeling comfortable leaving their house to go to an activity session. *Exercise specialist 3*

### Effectiveness and maintenance

Table 3 presents descriptive statistics for CLICK into Activity outcomes for each data collection point. Linear mixed models analyses revealed time to be a significant predictor of vigorous physical activity, moderate physical activity, walking, sport, total physical activity, sitting time, mental wellbeing, and grip strength (*p* < 0.001). Time and initial participation interactions were not found to be statistically significant (*p* > 0.05).

**Table 3:** Descriptive statistics: CLICK into Activity outcomes

Measure	Baseline (*N* = 602)			3 month follow-up (*N* = 186)			6 month follow-up (*N* = 80)			12 month follow-up (*N* = 41)		
	*N*	Mean	*SD*	*N*	Mean	*SD*	*N*	Mean	*SD*	*N*	Mean	*SD*
Vigorous PA^a^	600	0.65	4.71	184	58.59	68.28	79	82.41	76.89	41	53.66	80.89
Moderate PA^a^	599	10.11	19.88	184	127.20	162.15	79	82.41	76.78	41	72.32	79.28
Walking^a^	594	39.67	60.04	185	114.76	135.58	80	88.69	78.45	41	86.59	78.19
Total PA^a,b^	592	50.54	63.45	184	300.84	283.10	79	253.10	183.34	41	212.56	195.38
Sport^a^	598	0.20	2.31	185	19.76	33.82	78	21.35	43.48	41	21.83	42.60
Sitting time^a^	572	3092.48	1362.15	177	2296.36	985.43	75	2214.80	966.23	41	2053.90	838.05
Mental wellbeing^c^	485	47.74	10.88	177	53.90	9.94	76	57.25	8.46	40	58.35	10.47
Grip strength (Llbs)^d^	596	25.43	10.01	176	26.63	9.66	71	26.43	9.04	34	25.03	8.02

Numbers do not always sum up to total due to missing responses

*PA* physical activity

^a^ Minutes per week

^b^ Sum of physical activity data (vigorous PA, moderate PA and walking)

^c^ Total Warwick Edinburgh Mental Wellbeing Scale (WEMWBS) score out of a possible 70 points

^d^ Objectively-measured via dynamometer

As shown in Table 4, follow-up pairwise comparisons using Bonferroni adjustments revealed significant baseline to follow-up improvements in vigorous physical activity, moderate physical activity, walking, sport, total physical activity, sitting time, and mental wellbeing across each time point (*p* < 0.05). There was a significant improvement in grip strength from baseline to 3-month and baseline to 12-month follow-up (p < 0.05), but this change was not evident at 6-month follow-up (*p* = 0.15). Although measures were taken to reduce the effects of missing follow-up data in our analysis, [29] 6- and 12-month follow-up response rates were low and as such, these findings should be treated with caution.

**Table 4:** Effectiveness and Maintenance of CLICK into Activity: results from mixed linear models

	Estimate (change in minutes/week)	SE	*df*	*95% CI*
Vigorous PA				
Baseline to 3-month follow-up	58.59**	3.36	655.23	50.53, 66.66
Baseline to 6-month follow-up	62.24**	6.77	729.08	45.99, 78.49
Baseline to 12-month follow-up	55.87**	6.22	692.85	40.96, 70.79
Moderate PA				
Baseline to 3-month follow-up	116.99**	7.30	667.55	99.47, 134.51
Baseline to 6-month follow-up	50.88**	14.96	808.68	14.98, 86.78
Baseline to 12-month follow-up	64.34**	13.72	810.94	31.42, 97.27
Walking				
Baseline to 3-month follow-up	65.73**	6.51	487.88	50.08, 81.38
Baseline to 6-month follow-up	39.42*	14.19	694.91	5.36, 73.47
Baseline to 12-month follow-up	37.83*	13.30	805.96	5.92, 69.74
Total PA^a^				
Baseline to 3-month follow-up	234.87**	12.21	622.24	205.56, 264.185
Baseline to 6-month follow-up	151.56**	25.23	804.96	91.04, 212.09
Baseline to 12-month follow-up	158.60**	23.14	804.86	103.11, 214.10
Sport				
Baseline to 3-month follow-up	20.04**	1.72	15.92	15.92, 24.16
Baseline to 6-month follow-up	21.28**	3.66	12.50	12.50, 30.07
Baseline to 12-month follow-up	29.54**	3.38	21.44	21.44, 37.65
Sitting time				
Baseline to 3-month follow-up	− 726.14**	91.96	248.91	− 947.78, 504.50
Baseline to 6-month follow-up	− 711.54**	191.83	136.03	− 1176.52, − 246.56
Baseline to 12-month follow-up	− 632.73**	159.71	112.72	− 1020.86, − 244.59
	Estimate (change in score)	SE	*df*	*95% CI*
Mental wellbeing^b^				
Baseline to 3-month follow-up	5.56**	0.62	223.10	4.05, 7.06
Baseline to 6-month follow-up	6.33**	1.38	244.32	2.99, 9.66
Baseline to 12-month follow-up	9.09**	1.42	142.84	5.65, 12.53
Grip strength (Llbs)^c^				
Baseline to 3-month follow-up	1.59**	0.38	204.99	0.69, 2.50
Baseline to 6-month follow-up	1.58	0.80	175.44	−0.35, 3.51
Baseline to 12-month follow-up	2.15*	0.76	73.89	0.29, 4.00

*PA* physical activity. Models adjusted for age category (18–34 years, 35–50 years, 51–69 years, 70 years and above), sex (male/female), BMI category (25 and under, 26–29, 30 and above), GP surgery, and initial participation in at least one CLICK into Activity session (yes/no)

*= *p* < 0.05

**= *p* < 0.001

^a^ Sum of physical activity data (vigorous PA, moderate PA and walking)

^b^ Warwick Edinburgh Mental Wellbeing Scale (WEMWBS) score. WEMWBS is scored out of a possible 70 points

^c^ Objectively-measured via dynamometer

Qualitative interviews with CLICK into Activity participants were consistent with quantitative findings, in that numerous positive changes were identified in participants' outlook and perceptions of their health and wellbeing as a result of being referred to the programme. These included increased mobility, weight loss, reduced symptoms from long-term conditions, increased core strength, and increased purpose and feelings of happiness.

To sum it up in a sentence, it’s brought me back to life. It’s as if I have been in hibernation. I’m a lot happier, fitter, and I can do that little bit more...I’ve lost quite a lot of weight, and I am a happier, happier person since. It’s given me more hope and a more positive attitude. It just does me so much good. I even bought a t-shirt, believe it or not. It says, ‘I got CLICKed into Life’. *Participant 3*

At the end of the 12-week enrolment period, CLICK into Activity participants were signposted to a range of alternative local physical activity classes. Feedback from qualitative interviews indicated that these were best received when personally recommended by an exercise specialist, as they were seen to provide trusted advice. Data also suggested that participants held positive intentions to continue to participate in physical activity after completing the programme. Participants were asked to discuss their views on paying for a service such as CLICK into Activity in the future. Most participants accepted that for such a programme to be sustained a personal contribution towards running costs was to be expected. However, it was also acknowledged that most attendees were of pensionable age with limited disposable income, so subsidised rates would be welcomed.

At an organisational level, steering group members reflected upon the potential sustainability of the programme, and highlighted concerns about engagement from GP surgeries beyond project funding.

Everyone was very enthusiastic [about the GP referral process] at the beginning [of the project] but it’s tailed off and I think this is because of the pressures that the [GP] surgeries are under. People have stopped thinking about good ideas and prevention but just gone to fire-fighting mode. *General Practitioner*

Referral to physical activity sessions continued at seven of nine surgeries until the end of project funding, but maintenance of the programme from referral through to activity session delivery beyond this time was uncertain due to the challenging economic climate.

### Adoption

A total of nine GP surgeries adopted CLICK into Activity for at least some of the programme delivery period. Eight surgeries were originally invited to participate in the programme at the outset, but two were withdrawn due to low recruitment rates and low project buy-in; one 2-years into project delivery (November 2017) and the other in June 2018. In June 2017 a ninth GP surgery was invited to participate.

Interviews with members of the project steering group revealed that a surgery’s decision to adopt the CLICK into Activity programme was associated with a range of engagement activities with practice staff arranged in advance of programme sign-up and delivery. One example included a talk from an external General Practitioner with a specialism in physical activity; this was thought to highlight the importance of physical activity promotion, and thus increase buy-in at staff and setting levels. Qualitative interviews also identified the importance of the presence of a GP staff member visibly recommending and championing the programme in advance of programme adoption. For example, an interview with the CLICK into Activity project manager revealed that the practice manager of one GP surgery - the surgery that was invited to adopt the project following the withdrawal of two originally recruited surgeries - had lobbied for involvement with the project from its initiation and was highly enthusiastic about physical activity promotion. The CLICK into Activity project manager felt that without this enthusiasm from one member of the team, the surgery and its staff would not have agreed to adopt the programme.

### Implementation

Exercise specialists reported that a key influence on effective GP referral implementation was the presence of a practice manager or staff member who had an appreciation for the value of physical activity for patients with, or at-risk of a long-term condition. Notably, an assessment of programme documentation found that the late joining surgery referred 66 participants in 12 months (11.0% of total sample), while the two surgeries withdrawn from the project referred only 49 participants between them (8.1% of total sample). One of the exercise specialists expressed surprise at the attitudes of some health professionals at participating surgeries. It was noted that physical activity was not always seen as a priority prevention strategy, and that this had a negative impact on successful implementation.

I thought ‘Great I’m going to be a team with the doctors, we are going to really work together’. I thought doctors would know the benefits of exercise, but I was shocked to see that some of them needed educating. They didn’t believe in exercise. *Exercise specialist 1*

Reflections on physical activity session implementation revealed that exercise specialist characteristics were integral to success. Participants reported that exercise specialists created a safe and supportive environment, instilling confidence in them from initial consultation to the end of the programme. Support and guidance from exercise specialists to participate in appropriate tailored physical activity was perceived to promote increased feelings of control over participants’ health and wellbeing. Participants also reported feelings of increased self-worth and happiness because of their engagement with exercise specialists.

The only words to describe [the exercise specialist] are ‘excellent’ and ‘awesome’. She is very, very dedicated. She deserves a medal, literally. She is very, very good; she knows what she’s doing. *Participant 2*

Another important feature of programme implementation success was the group delivery of CLICK into Activity sessions. Programme sessions created an opportunity for social engagement with members of the local community with similar health profiles. Many participants reported feelings of social isolation prior to CLICK into Activity, which improved through meeting new people and building social support through the programme.

I think everybody was a bit nervous to start, and then as you got to know people and more and more people joined, the old [participants] were, like, helping the new [participants]. It was just amazing because everybody said they were so nervous, and the older [participants] made the new [participants] feel so welcome. *Participant 3*

Qualitative interviews with CLICK into Activity participants also revealed that the content of the physical activity sessions was important, with praise for circuit training activities tailored to individuals’ needs and abilities. In response to the popularity of circuit training sessions observed through attendance figures and anecdotal feedback provided to exercise specialists, the CLICK into Activity schedule was adjusted over time, with the provision of adapted sports sessions reduced in the final year of project delivery to allow additional capacity for circuit training.

The type of class was important. We did have adapted sports, like table tennis and then Boccia and ‘new age’ curling. Some of these are really good fun to attend but I think for this type of project [with inactive adults], the circuit style delivery is better and [it] was much more popular. I think this was because people felt they were getting more for their time. The numbers are picking up where adapted sports has been swapped for circuits. *Project manager*

[The exercise specialist] knows how to sort of treat us ‘older people’, in the fact that caution has to be adhered to. You know, you don’t want to push people too hard. *Participant 10*

Interviews revealed that successful implementation of the programme was reliant on good communication across all levels of programme delivery. It was reported that quarterly steering group meetings were not always well attended due to competing pressures on project partners’ time, but they were perceived to provide an opportunity to share good practice and draw upon expertise from those working in a different field but working toward the same objective.

As shown in Table 5, the total cost of CLICK into Activity implementation over 3 years was £174,396 (2017–18 prices), based on total annual delivery and preparation costs. The average cost per person attending at least one CLICK into Activity session was £535. Annual preparation cost estimates, including training expenses, were relatively consistent (~£3000–£4000 each year), while delivery costs were seen to reduce over time as the programme became more established. Research and infrastructure development costs, including IT infrastructure, and evaluation and research expenses totalled £67,500 over 3 years. These costs were excluded from the total cost of implementation, as they would not apply to mainstream implementation.

**Table 5:** CLICK into Activity costs and resources

	Nov 2015 - Oct 2016 £ actual	Nov 2016-Oct 2017 £ actual	Nov 2017 –Oct 2018 £ estimate
Delivery Cost Estimate			
Staff (Salaries two exercise specialists)	42,128	43,368	41,215
Equipment	4002	1723	1250
Hire of Facilities	27,063	26,696	25,000
Surgery Room Hire (in Kind)	−22,080	−22,080	−22,080
Promotion & Publicity	7358	3210	4000
Transport/Travel	441	360	500
Sub Total	58,912	53,277	49,885
Preparation Cost Estimate			
Training & Coaching fees/expenses	4871	4451	3000
Sub-total	4871	4451	3000
Research & Infrastructure Development			
IT Infrastructure	22,500	–	–
Evaluation & Research	12,500	16,250	16,250
Sub-total	35,000	16,250	16,250
Annual Cost of Implementation (Preparation and Delivery)	63,783	57,728	52,885
Total Cost of Implementation over 3 Years from a funder perspective^a^			£174,396
Average Cost per person based on at least one attendance at CLICK into Activity 12-week programme^b^			£535

^a^ Sum of delivery cost estimate and preparation cost estimate. Excludes research and infrastructure development costs. ^b^ The average cost per person attending at least one CLICK into Activity session = a / number of people attending at least one session (*N* = 326)

## Discussion

This study applied the RE-AIM framework to evaluate the individual and organisational impacts of GP referral of inactive adults living with (or at risk of) long-term conditions to community-based physical activity. The collection of quantitative and qualitative data from a range of sources, and the application of the RE-AIM framework, helped to identify the impacts of the programme while also highlighting potential barriers and facilitators to real-world implementation.

CLICK into Activity reached just under one third of the target population (30.2%). It is difficult to assess this figure against similar studies, as reported estimations of reach often fail to reflect the true number of eligible participants and those that go on to participate in an intervention [13, 16]. Recruitment figures were seen to increase following GP mail-outs, adding to the evidence base in support of utilising active recruitment strategies (e.g. health professional referral or targeted mail-out [35]) to engage a representative target audience for a physical activity programme [36]. Relaxation of the eligibility criteria to include obese and overweight individuals saw recruitment increase to some extent. However, recruitment was most actively impacted by GP mail-out. These findings highlight the importance of taking a flexible approach to marketing and recruitment to promote programme reach. Factors contributing to initial (non-)participation were numerous and varied, including individual-, social-, and environmental-level barriers and facilitators. These findings are consistent with those from a qualitative review of reasons for physical activity participation among children and adults [37]. Future programmes may need to consider and address some of the socio-ecological barriers preventing inactive adults from initial attendance, as our findings suggest that once an individual attends one session they often return.

Overall, positive quantitative and qualitative findings were found for programme effectiveness, and many positive outcomes were maintained up to 12 months. Follow-up physical activity responses exceeded UK physical activity recommendations for adults and older adults [38] and are particularly encouraging in light of UK Government targets to tackle inactivity [6, 7]. No differences were observed in target outcomes between those that attended a physical activity session and those that did not. No qualitative interviews were conducted with individuals that did not attend a session and therefore it is difficult to interpret this finding. However, this finding is consistent with those reported elsewhere [39, 40]. One possible explanation is that the GP referral process was a brief intervention in itself, encouraging individuals to seek out activity independently; this warrants further examination.

Programme adoption at GP surgeries was successful, with all nine surgeries invited taking up the programme. However, there was only partial adoption from two surgeries, which were withdrawn from the project. Consistent with findings from a recent systematic review, [9] our study identified a range of factors influencing programme adoption. For example, engagement activities designed to promote staff ‘buy in’ prior to programme adoption are recommended. It is possible that our findings are not generalisable, but they are consistent with those reported elsewhere [41–43].

Findings suggest that future programmes involving GP referral to physical activity should consider involving surgery staff in programme development, to identify physical activity champions to influence effective GP referral implementation when the programme is up-and-running. In terms of physical activity session implementation, consistent with previous reviews [13, 16] the support and guidance provided by programme deliverers (i.e. exercise specialists) was seen to be central to the positive changes observed in respondent outcomes. Exercise specialists were credited with providing a catalyst for change from ‘inactive’ to ‘active’, and for recognising the importance of providing instructions on how to perform physical exercises; an approach advocated in a recent systematic review of physical activity interventions for inactive adults [44].

While previous reviews have identified benefits associated with group-based physical activity interventions in general [16], this is one of the first known studies to lend support to group-based interventions targeted specifically at inactive individuals living with a long-term condition. This finding is encouraging given evidence for positive associations between social support and physical activity participation among adults [45, 46] and older adults, [47].

The total cost of implementing CLICK into Activity over 3 years was approximately £175,000, with an average cost per person attending at least one session of £535. Unfortunately there is a lack of reporting on implementation costs and resources associated with adults’ physical activity interventions, [16, 39, 48] making it difficult to compare our findings with those of similar studies. However, the estimated cost of programme implementation compares favourably with the direct costs of disease management and common health conditions related to physical inactivity (not including costs to other parts of the NHS and wider health and social care system) [4].

### Strengths and limitations

Strengths of the study include application of the RE-AIM framework, including collation and triangulation of qualitative and quantitative data from a variety of sources; overcoming challenges in the evaluation of physical activity programmes [22, 39, 49] and social prescribing schemes [41–43] that have been reported previously. Limitations of the study include a lack of control or comparison group. Secondly, we were unable to recruit individuals that never participated in a CLICK into Activity session to participate in a qualitative interview; this limits our understanding of the barriers influencing initial engagement and participation. Thirdly, while our statistical approach was chosen precisely to mitigate the effects of missing data [29], 6- and 12-month follow-up response rates were low, which limits understanding of longer term programme effects. Fourthly, physical activity outcomes were based on self-report data collected by trained exercise specialists. Given reports of positive rapport developed between participants and exercise specialists, it is possible that participants over-reported activity levels for social approval, as observed in previous studies [50, 51]. The collection of physiological data relating to participants’ long-term conditions (for example, HbA1c levels among those participants with type 2 diabetes) was beyond the remit of this evaluation. In the case of type 2 diabetes, studies have shown that physical activity can improve glycaemic control among diabetic populations [52] and it could reduce type 2 diabetes incidence [53]. Future studies examining physiological as well as physical and mental health outcomes would be beneficial.

## Conclusions

This study used the RE-AIM framework to evaluate the individual and organisational impacts of GP referral of inactive adults living with (or at risk of) long-term conditions to community-based physical activity. Although the target for programme reach was not met, and 6- and 12- month follow-up questionnaire responses were low, positive changes in physical activity and other outcomes assessed were observed among individuals that took part. Programme adoption at GP surgeries was successful, however, the GP referral process was not consistently implemented across participating surgeries. Physical activity sessions were successfully implemented; programme deliverers and group-based delivery were each identified as having an influential impact on programme outcomes, while changes to physical activity session content were made in response to participant feedback. An assessment of costs demonstrated the programme’s potential value for money. Overall, findings highlight strategies to be explored in future development and implementation of GP referral to community-based physical activity programmes targeting inactive adults living with (or at risk of) long-term conditions.

## Supplementary information

**Additional file 1.** CLICK into Activity logic model. This table is a logic model, presenting the programme delivery plan and hypothesised outcomes.

**Additional file 2.** Interview schedule for CLICK into Activity participants. This table presents an example interview schedule for CLICK into Activity participants.

**Additional file 3.** Consolidated criteria for reporting qualitative studies (COREQ). This table reports on the processes followed in undertaking the qualitative aspect of the research.

## Data Availability

This study does not have ethical approval to share the study dataset either in a repository or as supporting files. However, the data may be available from the corresponding author on reasonable request.

## Abbreviations

BMI: Body mass index
COREQ: Consolidated criteria for reporting qualitative research
GP: General practice
IPAQ: International Physical Activity Questionnaire
NHS: National Health Service
RE-AIM: Reach, effectiveness, adoption, implementation, maintenance
WEMWBS: Warwick Edinburgh Mental Wellbeing Scale
