# Supplementary material for: General practice referral of ‘at risk’ populations to community leisure services: applying the RE-AIM framework to evaluate the impact of a community-based physical activity programme for inactive adults with long-term conditions
Source: BMC Public Health. 2019 Oct 17;19:1308. doi: 10.1186/s12889-019-7701-5 (PMC6798368; doi:10.1186/s12889-019-7701-5)
Supplement: Supplementary file 1 — Additional file 1. CLICK into Activity logic model. This table is a logic model, presenting the programme delivery plan and hypothesised outcomes. [file 12889_2019_7701_MOESM1_ESM.docx]

Additional file 1. CLICK into Activity logic model

| **Situation / Need** | **Resources / Inputs** | **Activities** | **Outputs** | **Outcomes** | |
| --- | --- | --- | --- | --- | --- |
|  |  |  |  | **Short-term** | **Long-term** |
| No local provision GP referred physical activity meeting NICE guidelines for those with type 2 diabetes    Low levels of physical activity in the area    Rural setting in SW England – limited walkability and options for public transport | Nine participating GP surgeries    Designated space identified for exercise specialist consultations    Local organisations providing advice and support    Administration support from District Council    Funding for trained exercise specialists, project manager, evaluation support, programme activities (venue hire, equipment) and promotional work (e.g. flyers, reminder cards, posters, etc.) | Provision of 12-week GP referred physical activity programme in local community settings    Trained exercise specialist engagement with GP surgeries    GP referral to CLICK into Activity through GP/health care professional appointment or via direct mail out    Recruitment of eligible individuals to intervention via one-to-one screening consultation    Programme promotion and awareness-raising in local community    Quarterly steering group meetings with project stakeholders    Regular contact between exercise specialists, project manager and evaluation team | Number (and proportion) of target population that engage with the programme (attend screening consultation)    Number (and proportion) of target population that participate in the programme (attend programme)    Number of participants retained    Number of participants that move from ‘inactive’ to ‘active’    Number and types of community settings that participate    Number and types of staff that participate    Number and types of programme activities | Increased physical activity at individual and community levels    Improved mental wellbeing    Increased sense of community cohesion | Improved general health and wellbeing    Reduced burden on health and social care provision    Improved engagement in physical activity promotion among range of local stakeholders    Sustainable model for socially prescribed physical activity |
