# Supplementary material for: General practice referral of ‘at risk’ populations to community leisure services: applying the RE-AIM framework to evaluate the impact of a community-based physical activity programme for inactive adults with long-term conditions
Source: BMC Public Health. 2019 Oct 17;19:1308. doi: 10.1186/s12889-019-7701-5 (PMC6798368; doi:10.1186/s12889-019-7701-5)
Supplement: Supplementary file 2 — Additional file 2. Interview schedule for CLICK into Activity participants. This table presents an example interview schedule for CLICK into Activity participants. [file 12889_2019_7701_MOESM2_ESM.docx]

Additional file 2. Interview schedule for CLICK into Activity participants

| **Section** | **Questions** | **Timings** |
| --- | --- | --- |
| Introduction | Explain purpose of the interview   - *The reason we’d like to talk to you is because we think that you will be able to help us to improve CLICK into Activity in the future and we value your opinions. We are going to talk about your experience of taking part in the intervention and how you think the intervention would be best promoted in the future.*   Explain audio recording and data storage procedures   - *Before we get started I’d like to tell you that I will be recording the conversation to help us remember what we discussed. You can ask for the recording to be stopped at any time. After the interview the recording will be written up and we will remove any identifiable information like names, place names, etc. At this point audio files will be deleted so none of the information that is written down and recorded can be connected to you in any way.*   Position interviewee as the expert   - *There are no right or wrong answers. We are trying to understand your views on how the project worked – as you are the expert! Please be as honest as possible.*   Answer any questions  Researcher to complete verbal consent form with participant | 3 mins |
| Ice breaker | To start us thinking about your involvement in CLICK into Activity, can you tell me what attracted you to the project? | 2 mins |
| Barriers and facilitators to participation | Now I’d like us to start by finishing off some sentences. I will go through each sentence and I’d like to tell me how you would finish it. We’ll then think about your response to each sentence in a bit more detail.   - I enjoyed CLICK into Activity because… - I didn’t enjoy or like being part of CLICK into Activity because… - I found it easy to attend CLICK into Activity sessions because… - I found it difficult to come to CLICK into Activity sessions because…   More in-depth exploration of items above:   - Can you tell me a bit more about what you enjoyed/didn’t enjoy about CLICK into Activity? - Can you tell me a little more about why you found it easy/hard to attend sessions? - Do you have any specific examples that you can share?   PROMPTS   - Cost - Activity content - Day of session - Number of sessions - Length of sessions - Family commitments - Weather | 15 mins |
| Session experiences | Did you get to know people during the 12-week programme?  Did your relationship with people change as the weeks went by?  Were people supportive? Do you have any specific examples?  How did you feel during the sessions physically (e.g., hot, sweaty, out of breath)?  Did the sessions become easier over time?  Did you develop any skills during the 12-week programme?  What can you do now that you couldn’t before?  Has your confidence changed? In what way(s)?  How would describe the attendance at the sessions (e.g., high, low, variable)?  Did it change over the 12-week period?  Do you have any thoughts about why attendance was like it was?  How did attendance make you feel (e.g., did you feel better when there were more or less people?)? | 15 mins |
| Data collection | What we your experiences of the data collection process?  How could the process of data collection be improved? | 5 mins |
| Exercise specialists | Initial consultation   - How did you find your initial consultation (e.g., supportive, nerve-wracking, judgemental)? - Would you change anything about your initial consultation?   Overall impressions   - What did you think about your exercise specialist? - Is there anything you would change about the style of your exercise specialist? - Did your exercise specialist give you choices (e.g., different activities)? If yes, did you want this choice? - Do you think you had control over the activities you were involved with?   PROMPTS   - Things liked/liked less - Teaching style (e.g., encouraging, motivational, enthusiastic, good knowledge, made sessions too difficult, didn’t consider my needs) | 10 mins |
| Signposting | What did you think of the information you were given about local opportunities to continue physical activity?  Are you thinking of starting a new class now that CLICK into Activity has finished?  Have you already started a new class?  Did your exercise specialist advise you on other local activities/clubs in your area? | 5 mins |
| Roll out of the programme | We’d now like to know what you would change or what you think we should change if we were to do the project again.  If we were to run the programme again would you be willing to attend?  How much would you be willing to pay?  How could we make sessions more fun?  Do you think running this project on a larger scale would work? Why/why not? | 5 mins |
| Closing | Finally, is there anything that we have not discussed today that you think could have been done to improve CLICK into Activity?  Thank participant.  Provide opportunity for any additional information.   - That’s all the questions I have for you today. - Is there anything else you’d like to add?   Provide opportunity for exercise specialist to ask any questions.   - Do you have any questions for me? | 2 mins |
