# Supplementary material for: General practice referral of ‘at risk’ populations to community leisure services: applying the RE-AIM framework to evaluate the impact of a community-based physical activity programme for inactive adults with long-term conditions
Source: BMC Public Health. 2019 Oct 17;19:1308. doi: 10.1186/s12889-019-7701-5 (PMC6798368; doi:10.1186/s12889-019-7701-5)
Supplement: Supplementary file 3 — Additional file 3. Consolidated criteria for reporting qualitative studies (COREQ). This table reports on the processes followed in undertaking the qualitative aspect of the research. [file 12889_2019_7701_MOESM3_ESM.docx]

Additional file 3. Consolidated criteria for reporting qualitative studies (COREQ)

| **No. Item** | **Guide questions/description** | **Reported on Page #** |
| --- | --- | --- |
| **Domain 1: Research team and reﬂexivity** |  |  |
| *Personal Characteristics* |  |  |
| 1. Interviewer/facilitator | Which author/s conducted the interview or focus group? **ELB, MK.** | 11 |
| 2. Credentials | What were the researcher’s credentials? E.g. PhD, MD **MSc, MSc** | N/A |
| 3. Occupation | What was their occupation at the time of the study? **Senior Lecturer, Research Associate** | 28 |
| 4. Gender | Was the researcher male or female? **Female, female** | N/A |
| 5. Experience and training | What experience or training did the researcher have? **Both undertook formal training on qualitative research methods from MSc.** | N/A |
| *Relationship with participants* |  |  |
| 6. Relationship established | Was a relationship established prior to study commencement? **No** | N/A |
| 7. Participant knowledge of the interviewer | What did the participants know about the researcher? e.g. personal goals, reasons for doing the research **Both ELB and MK had met organisational-level stakeholders and exercise specialists on several occasions. CLICK into Activity recipients were unknown to ELB and MK but were informed about the reasons for doing the research at the start of the first interview.** | N/A |
| 8. Interviewer characteristics | What characteristics were reported about the interviewer/facilitator? e.g. Bias, assumptions, reasons and interests in the research topic **None** | N/A |
| **Domain 2: study design** |  |  |
| *Theoretical framework* |  |  |
| 9. Methodological orientation and Theory | What methodological orientation was stated to underpin the study? e.g. grounded theory, discourse analysis, ethnography, phenomenology, content analysis **Qualitative analysis was conducted using a thematic analysis.** | 12 |
| *Participant selection* |  |  |
| 10. Sampling | How were participants selected? e.g. purposive, convenience, consecutive, snowball **Purposive sampling for interviews.** | 9 |
| 11. Method of approach | How were participants approached? e.g. face-to-face, telephone, mail, email **Organisational stakeholders and exercise specialists were approached face-to-face. CLICK participants were approached via telephone after ticking a box in a questionnaire consenting to be contacted.** | 9 |
| 12. Sample size | How many participants were in the study? **Two semi-structured interviews were conducted with 10 CLICK into Activity participants (N=19). One CLICK participant did not complete follow-up due to relocation. Three exercise specialists (N=6). Two organisational stakeholders took part in a semi-structured interview (N=2). Total N = 27.** | 13/14 |
| 13. Non-participation | How many people refused to participate or dropped out? Reasons? **One participant did not complete follow-up interview due to relocation.** | 14 |
| *Setting* |  |  |
| 14. Setting of data collection | Where was the data collected? e.g. home, clinic, workplace **All interviews were conducted over the phone.** | 9 |
| 15. Presence of non-participants | Was anyone else present besides the participants and researchers? **No.** | N/A |
| 16. Description of sample | What are the important characteristics of the sample? e.g. demographic data, date **CLICK into Activity participants – all attended at least one session of CLICK into Activity.** | 13/14 |
| *Data collection* |  |  |
| 17. Interview guide | Were questions, prompts, guides provided by the authors? Was it pilot tested? **Yes. No.** | 10 and additional file 2 |
| 18. Repeat interviews | Were repeat interviews carried out? If yes, how many? **Yes. 13 of the 27 interviews were follow-up interviews.** | N/A |
| 19. Audio/visual recording | Did the research use audio or visual recording to collect the data? **Audio recordings were made of each interview.** | 12 |
| 20. Field notes | Were ﬁeld notes made during and/or after the interview or focus group? **No.** | N/A |
| 21. Duration | What was the duration of the inter views or focus group? **30 minutes.** | 9 |
| 22. Data saturation | Was data saturation discussed? **Yes, ELB, MK and Principal Investigator JP discussed findings and data saturation.** | 12 |
| 23. Transcripts returned | Were transcripts returned to participants for comment and/or correction? **No.** | N/A |
| **Domain 3: analysis and ﬁndings** |  |  |
| *Data analysis* |  |  |
| 24. Number of data coders | How many data coders coded the data? **ELB and MK. Final themes were agreed by authors.** | 12 |
| 25. Description of the coding tree | Did authors provide a description of the coding tree? **No.** | N/A |
| 26. Derivation of themes | Were themes identiﬁed in advance or derived from the data? **Data were explored using thematic analysis,[28] with the coding process based predominantly on mapping data against each of the RE-AIM dimensions in line with recently published guidance[29]. Analysis aimed to generate a balanced assessment of the programme and the factors that may have had an impact on the reach, effectiveness, adoption, implementation and potential sustainability of CLICK into Activity** | 12 |
| 27. Software | What software, if applicable, was used to manage the data? **NVivo 10.** | 12 |
| 28. Participant checking | Did participants provide feedback on the ﬁndings? **No.** | N/A |
| *Reporting* |  |  |
| 29. Quotations presented | Were participant quotations presented to illustrate the themes/ﬁndings? Was each quotation identiﬁed? e.g. participant number **Yes. Yes.** | 13-22 |
| 30. Data and ﬁndings consistent | Was there consistency between the data presented and the ﬁndings? **Yes.** | N/A |
| 31. Clarity of major themes | Were major themes clearly presented in the ﬁndings? **Yes.** | 15-21 |
| 32. Clarity of minor themes | Is there a description of diverse cases or discussion of minor themes? **Yes.** | 15-21 |
